# Pericardial Effusion due to Primary Malignant Pericardial Mesothelioma: A Common Finding but an Uncommon Cause

**DOI:** 10.1155/2016/4810901

**Published:** 2016-11-28

**Authors:** Valery Istomin, David S. Blondheim, Simcha R. Meisel, Aaron Frimerman, Moshe Lapidot, Ronit Rachmilevitch

**Affiliations:** ^1^Department of Internal Medicine C, Hillel Yaffe Medical Center, Hadera, Affiliated to Technion, Haifa, Israel; ^2^Department of Cardiology, Hillel Yaffe Medical Center, Hadera, Affiliated to Technion, Haifa, Israel; ^3^Department of Thoracic Surgery, Rambam Medical Center Affiliated to Technion, Haifa, Israel

## Abstract

This case report describes a 37-year-old female who was admitted to our Emergency Department because of shortness of breath. On physical examination, she had dyspnea and tachycardia and blood pressure was 80/50 mmHg with a pulsus paradoxus of 22 mmHg. Neck veins were distended, heart sounds were distant, and dullness was found on both lung bases. Her chest X-ray revealed bilateral pleural effusion and cardiomegaly. On both computed tomography and echocardiography the heart was of normal size and a large pericardial effusion was noted. The echocardiogram showed signs of impending tamponade, so the patient underwent an emergent pericardiocentesis. No infectious etiology was found and she was assumed to have viral pericarditis and was treated accordingly. However, when the pericardial effusion recurred and empirical therapy for tuberculosis failed, a pericardial window was performed. A typical staining pattern for mesothelioma was found on her pericardial biopsy specimen. Since no other mesodermal tissue was affected, a diagnosis of primary malignant pericardial mesothelioma was made. Chemotherapy was not effective and she passed away a year after the diagnosis was made. This case highlights the difficulties in diagnosing this uncommon disease in patients that present with the common finding of pericardial effusion.

## 1. Introduction

Pericardial effusion is a common finding in clinical practice either as an incidental finding or as a manifestation of a systemic or cardiac disease. In the current communication, we report on a young woman with recurrent cardiac tamponade due to an extremely rare disease, primary malignant pericardial mesothelioma (PMPM). Malignant mesothelioma is a highly invasive tumor arising from mesothelial cells of pleura, peritoneum, pericardium, or tunica vaginalis of the testis. The reported incidence of this tumor is approximately one per million. Pleural involvement is present in 88.8% of cases and peritoneal and testicular involvement in 9.6% and 0.2%, respectively. The incidence of PMPM was 0.002% in an autopsy series of 500,000 cases [1]. Although PMPM constitutes only 0.7% of all mesotheliomas, it is the most common primary pericardial tumor and accounts for about half of them [2]. In contrast to pleural mesothelioma, the association between PMPM and exposure to asbestos has not been confirmed.

## 2. Patient Description

**Figure 1:**
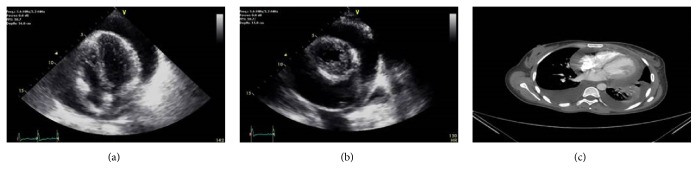
Transthoracic echocardiography demonstrates a large pericardial effusion ((a) apical 4-chamber view and (b) parasternal short axis view) and chest CT shows a large pericardial effusion, bilateral pleural effusions, and atelectasis of the left lower lobe (c).

A 37-year-old female was admitted with new onset of shortness of breath. On physical examination she was dyspnoeic, heart rate was 110 beats/min, and blood pressure was 80/50 mmHg with a pulsus paradoxus of 22 mmHg. Neck veins were distended; heart sounds were distant; no pericardial friction rub was heard; dullness was found on both lung bases. Chest X-ray revealed bilateral pleural effusions and severe cardiomegaly. On electrocardiogram (ECG), low voltage and widespread ST segment elevations were found. Echocardiography demonstrated a large pericardial effusion (Figures 1(a) and 1(b)) with collapse of the right atrium and ventricle as well as classical hemodynamic changes, typical of tamponade.

On emergent pericardiocentesis, 770 cc of hemorrhagic fluid was aspirated. Cultures of the fluid were sterile, and neither acid fast bacilli nor malignant cells were found. On chemical analysis of the pericardial fluid a low glucose level of 3 mg% (normal: 60–80 mg%), a high LDH level of 6644 mg% (normal: usually <400 mg%), and 300 eosinophils/mL (17%, no normal values but probably <1%) were found. Pharyngeal and rectal cultures were negative for enteroviruses; serological tests for cytomegalic virus, Epstein Barr virus, Q-fever, and mycoplasma were negative; rheumatoid factor, anti-nuclear antibodies, anti-neutrophil cytoplasmic antibodies, and thyroid function tests were within normal limits. Computed tomography (CT) of the chest and abdomen was normal except for pericardial and bilateral pleural effusions and left lower lobe atelectasis (Figure 1(c)). Neither pericardial nor pleural calcifications or masses were seen. Benign pericarditis was suspected and the patient was treated with ibuprofen and colchicine for 16 weeks.

Three months later, the patient had recurrent dyspnea and clinical signs of cardiac tamponade and a large pericardial effusion was found on echocardiography. On repeat pericardiocentesis 1200 cc of sterile hemorrhagic fluid was aspirated. Tests for acid fast bacilli were again negative and on cytologic examination only reactive mesothelial cells were seen. Chemical characteristics of the pericardial fluid were similar to the previous analysis.

**Figure 2:**
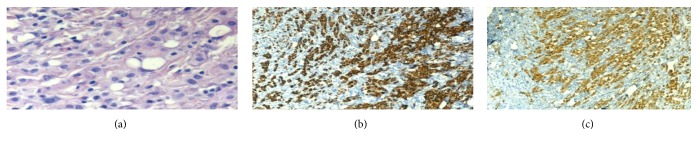
Histological examination of pericardium. The tumor cells are large polygonal cells with prominent nuclei and cytoplasmic vacuoles (Hematoxylin and Eosin staining, ×40) (a). Strong diffuse staining of mesothelioma cells with Cytokeratin 5/6 (×10) (b). Calretinin staining is strongly positive in tumor cells (×10) (c).

Since the patient immigrated to Israel from Ethiopia where TB is endemic and since she had hemorrhagic pericardial effusions with very low glucose levels and in the absence of a response to anti-inflammatory drugs, TB pericarditis was highly suspected and empiric antituberculous therapy was added to colchicine and prednisone. However, pericardial fluid reaccumulated during the following 4 weeks. An exploratory thoracoscopy and a pericardial window operation were performed and a pericardial biopsy was taken which showed large polygonal tumor cells with prominent nuclei and cytoplasmic vacuoles on Hematoxylin and Eosin staining. Cytokeratin and Calretinin staining were strongly positive in tumor cells, consistent with primary malignant mesothelioma. Subtotal pericardiectomy was performed. Tumor foci were also detected on histological examination of mediastinal fat (Figures 2(a), 2(b), and 2(c)). Since the pericardium was the only tissue originating from mesothelial cells in which malignant cells were found, a diagnosis of primary malignant pericardial mesothelioma (PMPM) was made. The patient denied any known contact with asbestos.

The patient was asymptomatic for a couple of months but on followup echocardiograms progressive encasement of the heart in tumor tissue was evident. Despite chemotherapy, a year later, the patient died.

## 3. Comment

The diagnosis of PMPM is a difficult one to make because its clinical picture is nonspecific: right sided heart failure due to cardiac tamponade or to constrictive pericarditis. Unfortunately, 70% of these tumors are diagnosed postmortem. Mass formation is not always present and thus neither echocardiography nor radiology is diagnostically helpful, as in the early stages of our case. Pericardiocentesis is diagnostic in only 25% of cases, explaining the lack of malignant cells on both cytological examinations of the pericardial fluid, and a pericardial biopsy is often required to make the correct diagnosis.

Median survival of PMPM from symptom onset is 6 months [3]. This poor prognosis is probably secondary to late diagnosis, incomplete tumor resection, and poor response to radiation and chemotherapy. Randomized trials in patients with pleural mesothelioma have shown a significant survival benefit, higher response rates, and a longer remission when treated with a combination of new antifolates and platinum-based chemotherapy, after excision of the tumor or with addition of bevacizumab in patients who were not candidates for curative surgery [4–6]. To the best of our knowledge, no reliable clinical trials of chemotherapy or radiotherapy for PMPM have been conducted. Some case reports demonstrate increased survival in patients with PMPM treated by combined chemotherapy [7]. Reardon et al. described a patient who did not respond favorably to chemotherapy after surgery but had a prolonged positive clinical response following high dose radiation [8].

In conclusion, PMPM should be included in the differential diagnosis of large pericardial effusions and cardiac tamponade and should be suspected in cases of refractory pericardial effusion despite appropriate anti-inflammatory treatment. In such patients pericardial biopsy should be performed as soon as possible. The diagnosis of PMPM is elusive because of the absence of previous exposure to asbestos, no tumor detection on radiologic and echocardiographic imaging, and negative cytological examinations of pericardial fluid. However, early diagnosis, appropriate surgery, and new chemotherapeutic and radiologic regimens may prolong survival in PMPM.
